# Supplementary material for: Dissecting Melanoma Ecosystem Heterogeneity from Molecular Characteristics to Genetic Variation at Single-Cell Resolution
Source: Int J Mol Sci. 2025 Oct 13;26(20):9956. doi: 10.3390/ijms26209956 (PMC12562883; doi:10.3390/ijms26209956)
Supplement: Supplementary file 1 [file ijms-26-09956-s001.zip › Supplemental Figures.pdf]

Supplemental Figures
Figures S1–S11

Figure S1

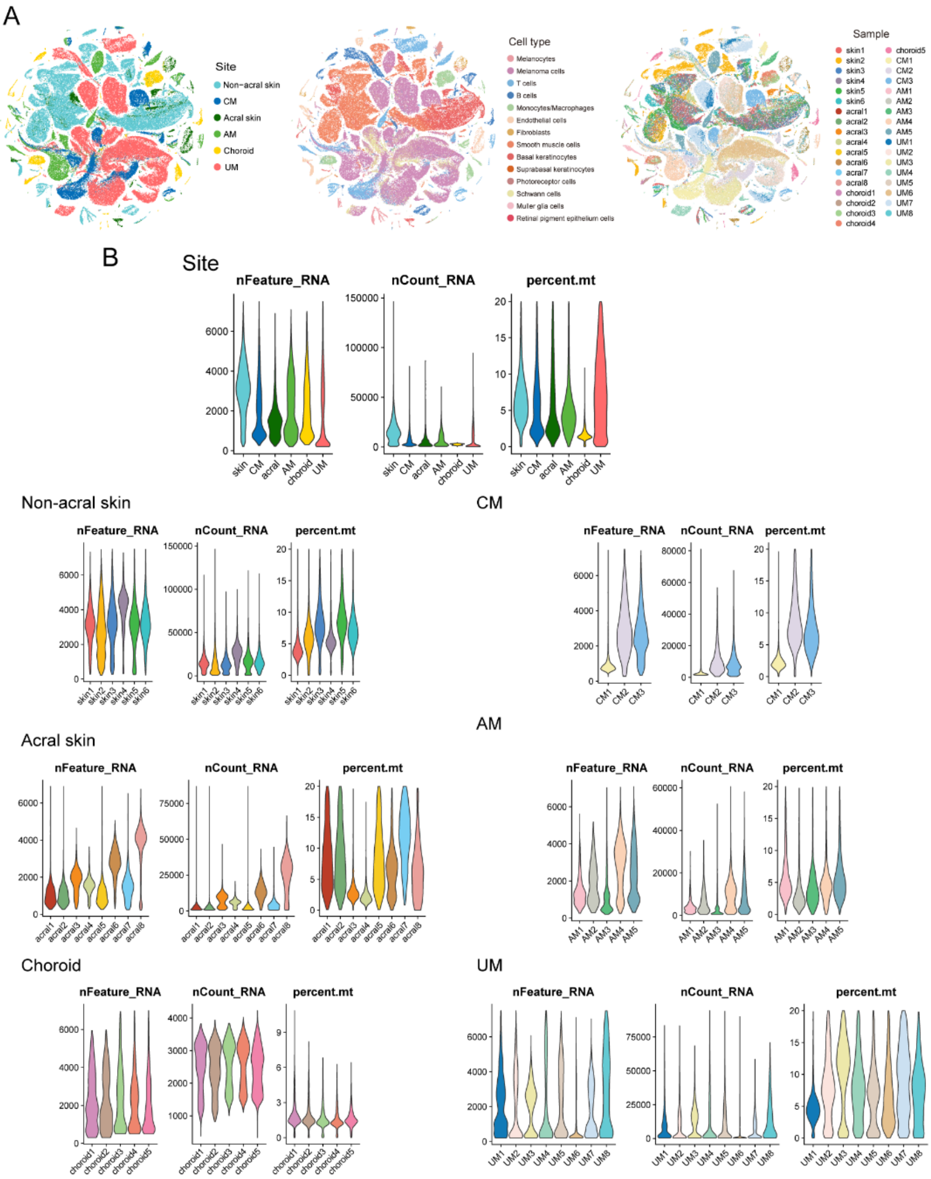

Figure S1 Single cell landscape and quality control in each melanoma site. **A** t-SNE projection showing cellular landscapes of all cells in each melanoma site, color-coded by sites (left panel), color-coded by cell types (middle panel) and color-coded by samples (right panel). **B** Violin plots showing quality control metrics of scRNA-seq data for high-quality in each melanoma sites.

Figure S2

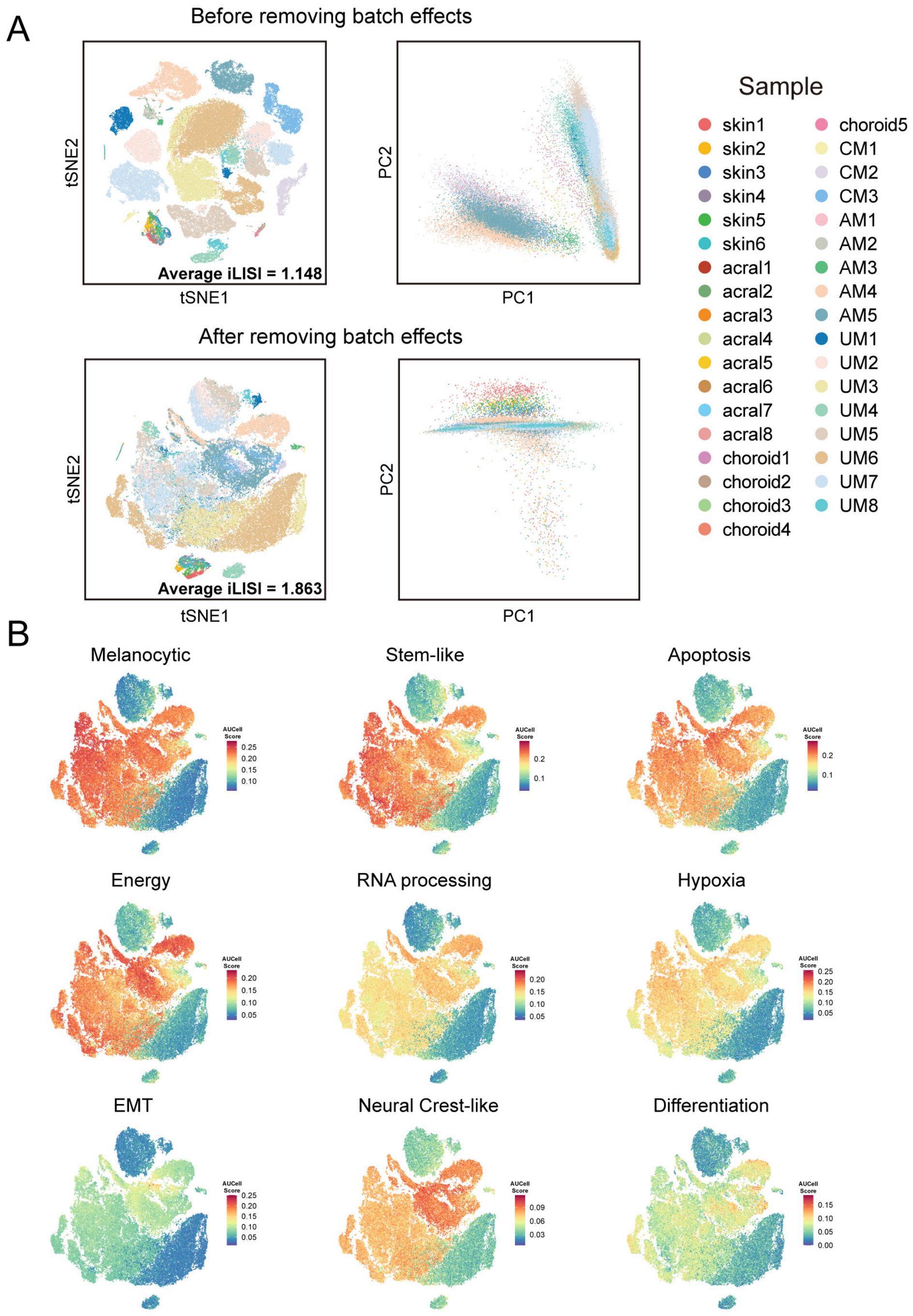

Figure S2 Batch correction of melanoma cells and biological signatures. **A** t-SNE projection showing batch correction of melanoma cell. Before batch correction of melanoma cells, there was an obvious batch effect between samples, and the cells clustered according to the samples (top panel, Average iLISI = 1.148). Batch correction significantly eliminates the batch effect of melanoma tumor cells (bottom panel, Average iLISI = 1.863), indicating minimal batch effects in melanoma tumor cell. **B** Feature plots show 9 signatures of melanoma cells.

Figure S3

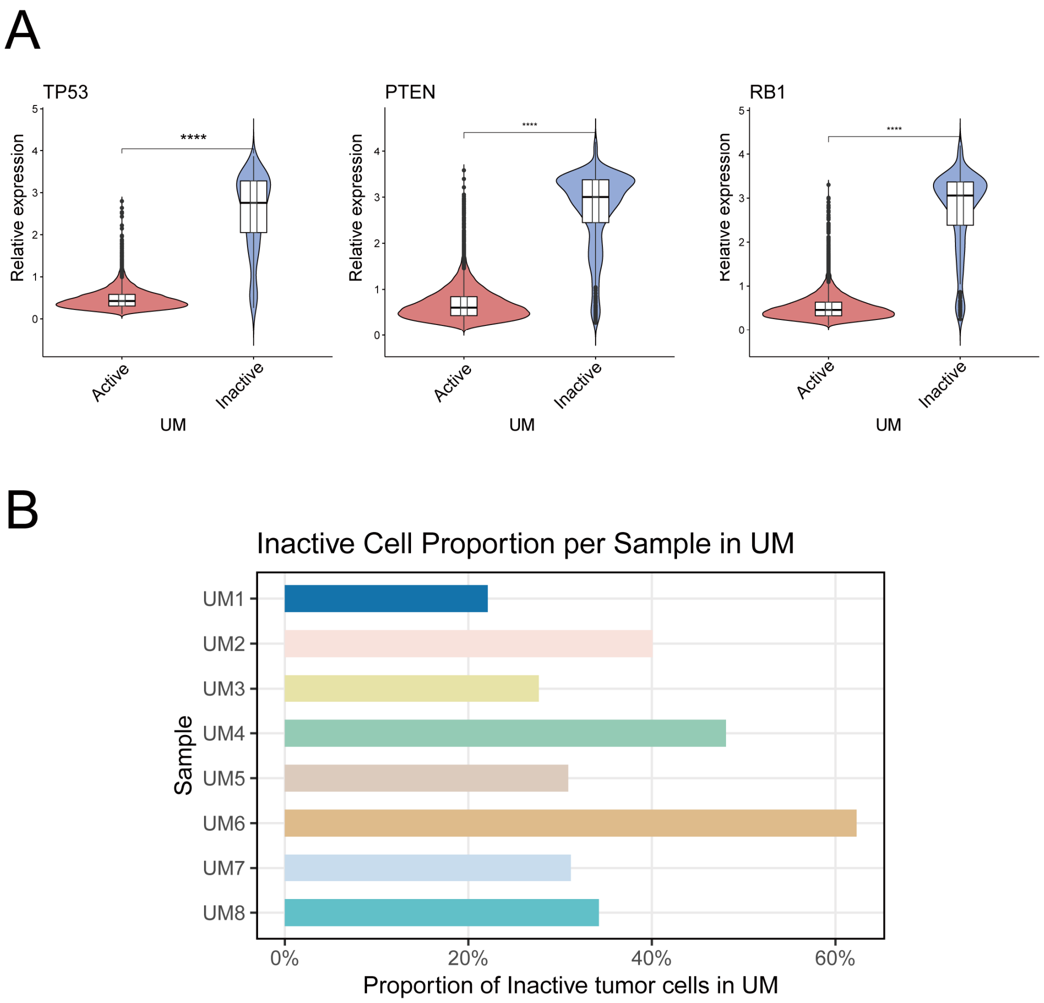

Figure S3 Tumor suppressor gene expression and abundance of inactive cells in UM. A Violin plots showing the expression of TP53, PTEN, and RB1 in the inactive and active cells of UM. Significance was determined using a two-sided, unpaired Wilcoxon rank-sum test, p-value denoted as *, ****p <= 0.0001. **B** Bar plot showing the proportion of inactive cells within each UM sample, calculated as the percentage of inactive cells relative to all melanoma cells in that sample.

Figure S4

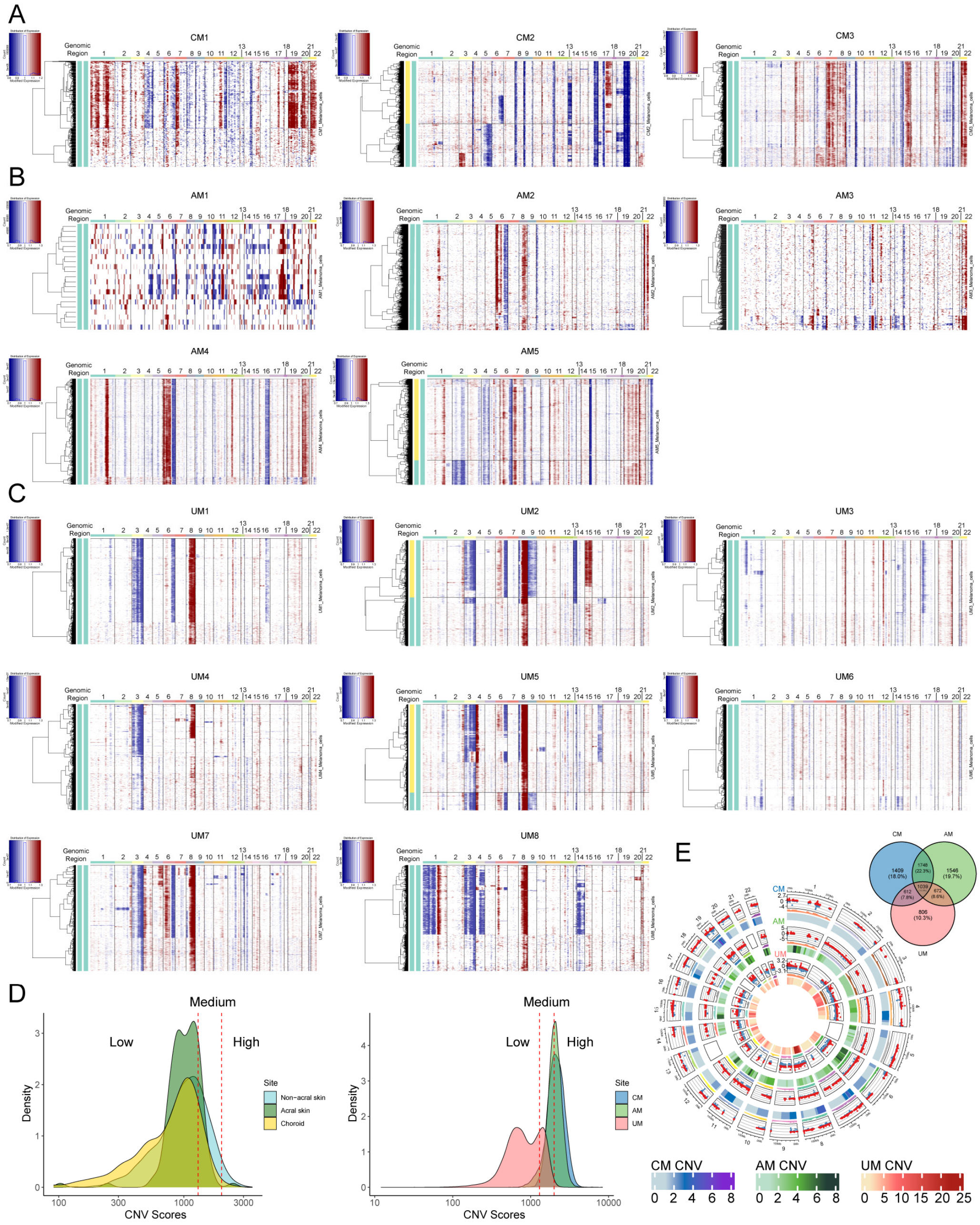

Figure S4 CNV of melanoma tumor cells in each melanoma site. A-C The hierarchical heatmap showing representative CNVs of melanoma tumor cell in each melanoma sample from CM (A), AM (B) and UM (C). **D** The cell density distribution of normal melanocytes (left panel) and melanoma cells (right panel) are categorized by their CNV scores into low, medium, and high CNV levels. **E** Circos plot showing differential genes with CNV events in each melanoma site. Color indicate score of CNV events in genes. Comparing melanoma cells and normal melanocytes, red dots represent gene upregulation ($\log_2$ fold change > 0) and blue dots represent gene downregulation ($\log_2$ fold change < 0) in melanoma cells. Venn diagram of all differential genes with CNVs events in CM, AM and UM.

Figure S5

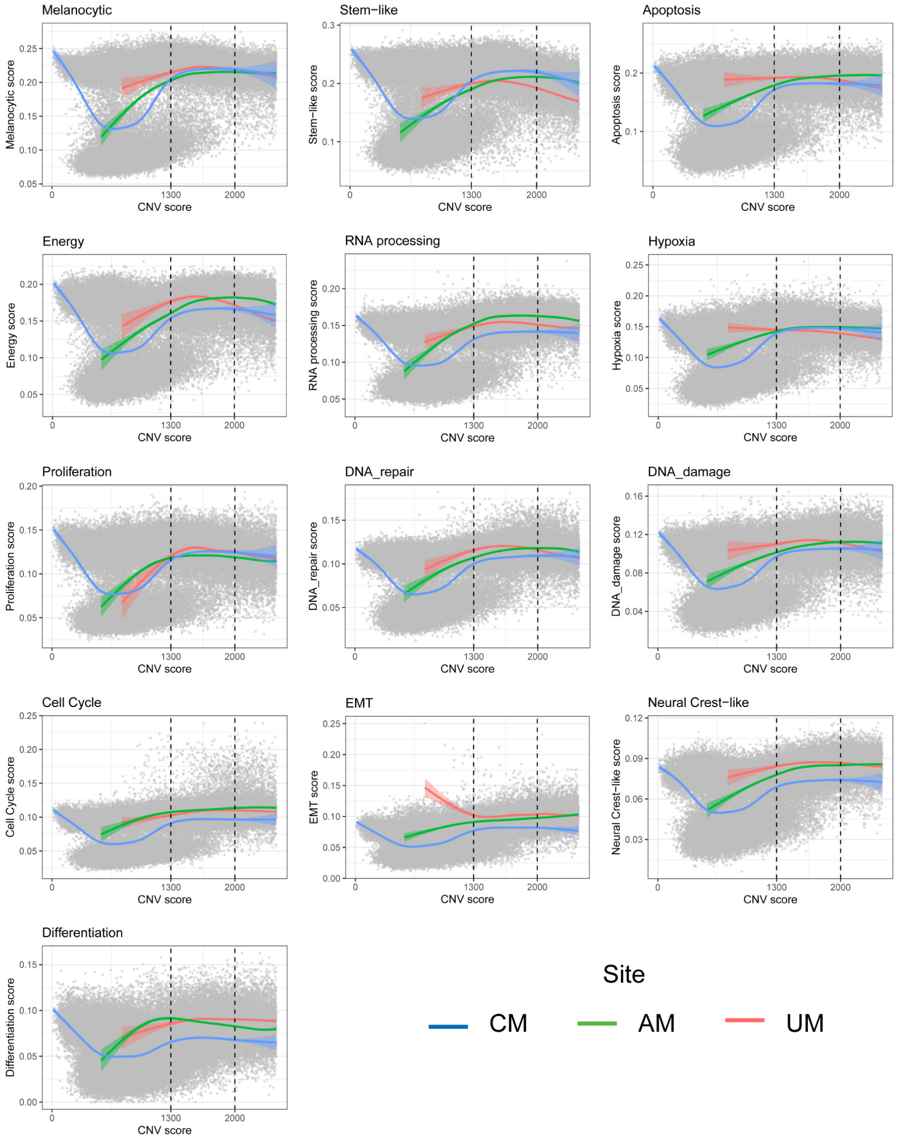

Figure S5 Association between AUCell score of melanoma signatures and CNV score changes in melanoma cells in each melanoma site.

Figure S6

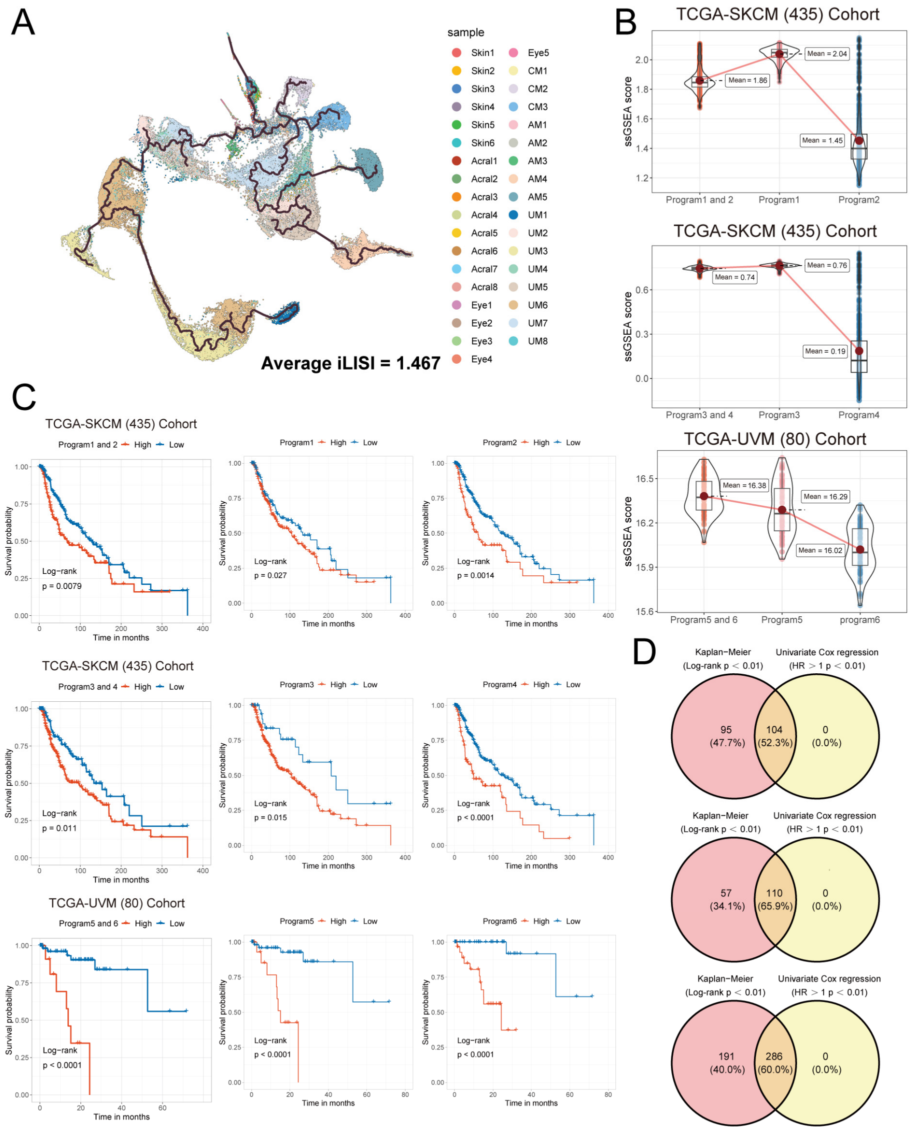

Figure S6 Batch effect evaluation of melanoma cell pseudotime trajectories and survival analysis of trajectory program genes

A Pseudotime analysis of melanoma cells from each melanoma site, color-coded by sample. Batch effect evaluation shows an average iLISI of 1.467, indicating minimal batch effects in the trajectory. **B** Violin plots showing the ssGSEA score of trajectory program gene in TCGA cohort patients. **C** Kaplan–Meier analysis showing the overall survival rate of TCGA cohort patients, characterized by high (red) and low (blue) levels of trajectory program gene expression. Significance was determined using a two-sided log-rank test. **D** Venn diagram of Kaplan–Meier analysis and Univariate Cox regression analysis of trajectory program gene in CM (top), AM (middle) and UM (bottom), intersection genes are trajectory program genes with survival prognostic response.

Figure S7

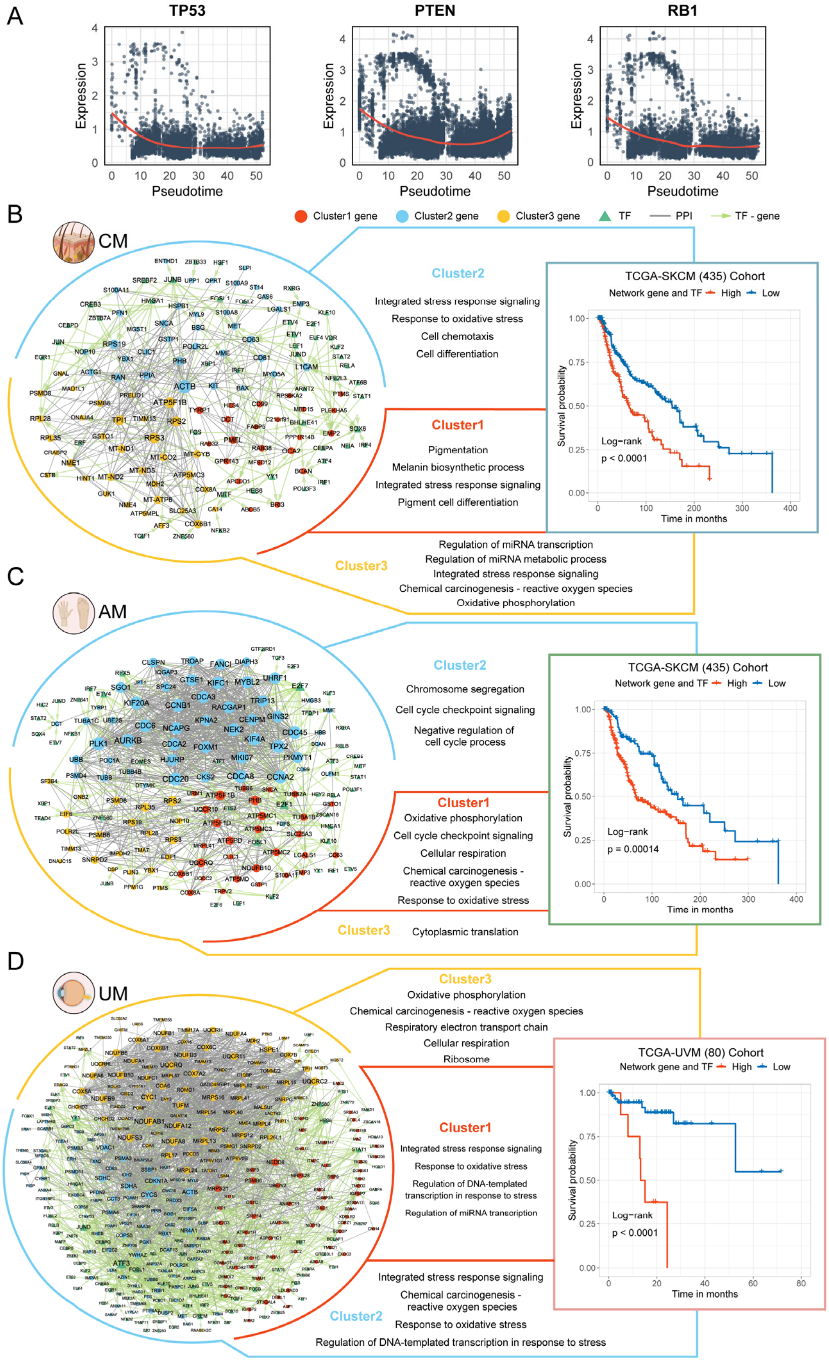

Figure S7 Expression patterns of critical tumor suppressor genes in UM and melanoma tumor cell "trajectory program gene -TFs" survival prognostic response network. **A** Expression patterns of critical tumor suppressor genes (TP53, PTEN, and RB1) along the pseudotime trajectory of uveal melanoma (UM) tumor cells. **B-D** "trajectory program gene-TFs" survival prognostic response networks, K-mean clusters and molecular characterization in CM (**B**), AM (**C**) and UM (**D**) melanoma tumor cell. Kaplan-Meier analysis demonstrates the relationship between network gene/TF and overall survival in melanoma patients, with significance determined by the two-sided log-rank test.

Figure S8

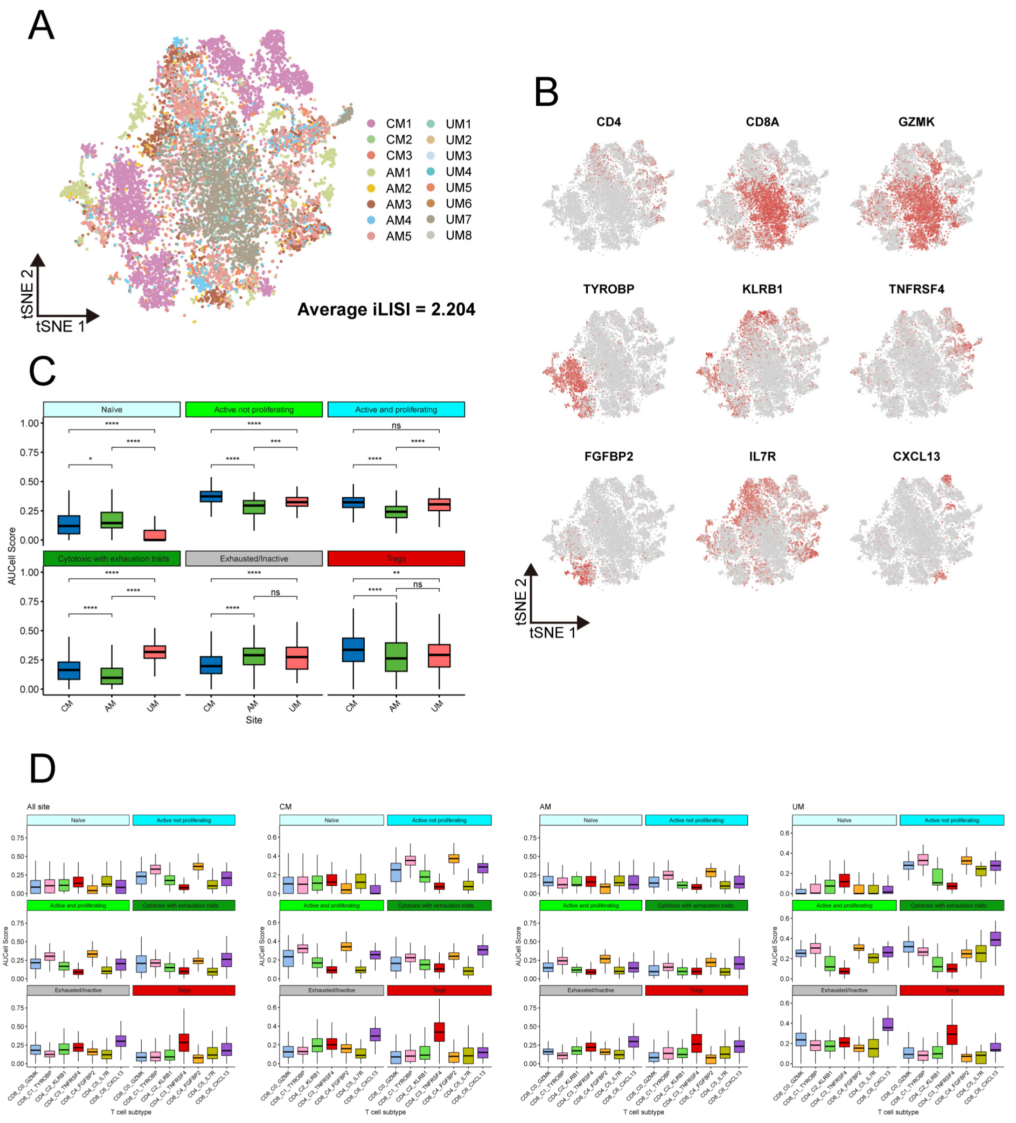

Figure S8 AUCell score of T cell subgroup states. A t-SNE projection showing T cell in samples, batch effect evaluation shows an average iLISI of 2.204, indicating minimal batch effects in the T cell subgroup. **B** Feature plots showing marker genes used for the annotation of T cell subgroups. **C** Boxplots showing the AUCell score of T cell states in each melanoma site. Significance was determined using a two-sided, unpaired Wilcoxon rank-sum test, *p*-value denoted as *, ns > 0.05, **p* <= 0.05, ***p* <= 0.01, ****p* <= 0.001, *****p* <= 0.0001. **D** Boxplots showing the AUCell score of T cell subgroup states in all site, CM, AM and UM.

Figure S9

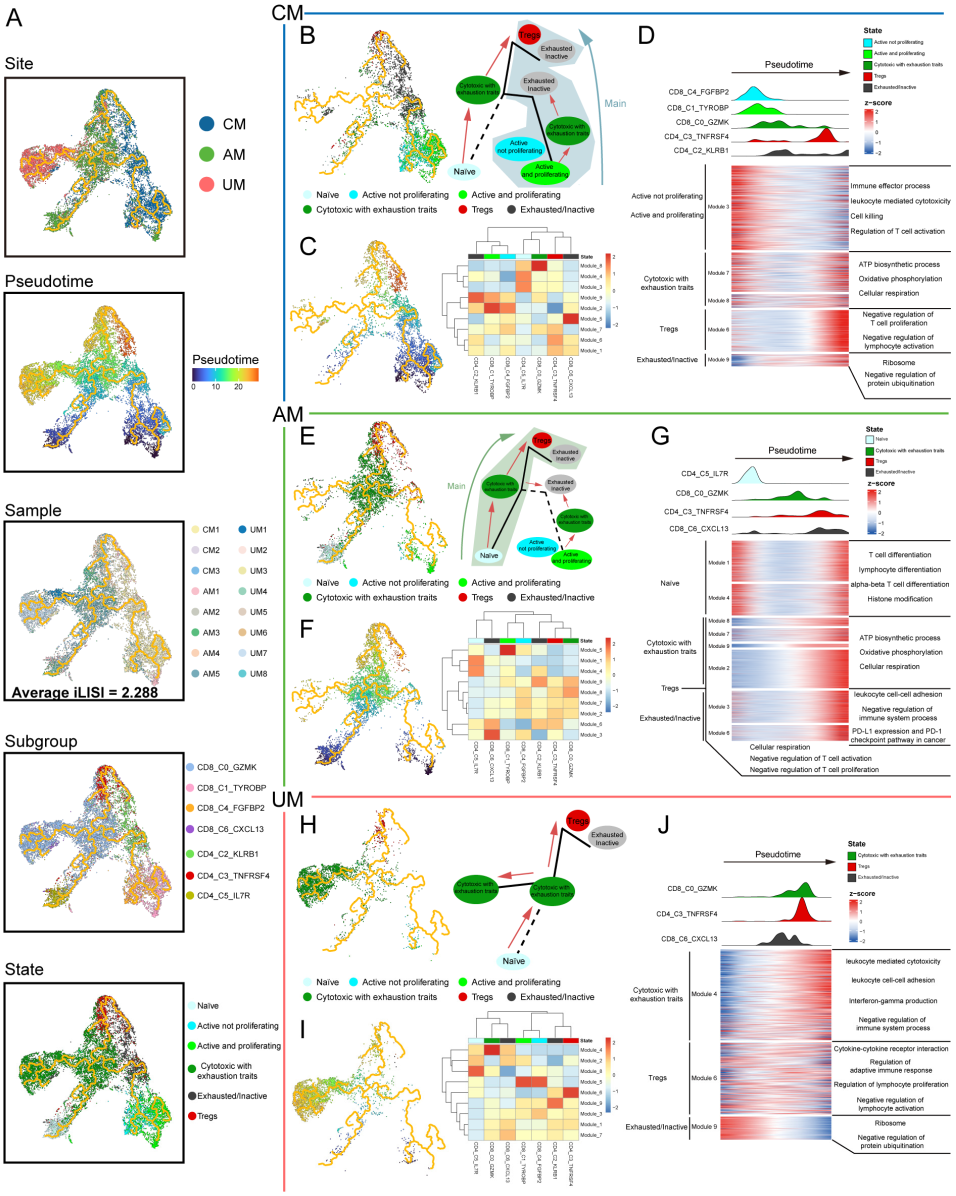

Figure S9 Trajectory analysis of T cell in each melanoma site. A Pseudotime analysis of T cells in each melanoma site. Color-coded by site, pseudotime, sample, subgroup and state. Batch effect evaluation shows an average iLISI of 2.288, indicating minimal batch effects in the trajectory. **B, E and H** Trajectory of T cell in CM (**B**), AM (**E**) and UM (**H**), the solid line indicates the potential major evolutionary direction in the trajectory, dashed line indicates the potential secondary evolutionary direction. **C, F and I** Pseudotime analysis of T cells in CM (**C**), AM (**F**) and UM (**I**) trajectories, color-coded by pseudotime (left panel). Heatmap showing gene modules of T cell subgroups in CM (**C**), AM (**F**) and UM (**I**) trajectories (right panel). **D, G and J** Heatmap showing the dynamic changes in major trajectory of gene expression along the pseudotime (bottom panel). The distribution of T subgroups during the transition along with the pseudotime (top panel).

Figure S10

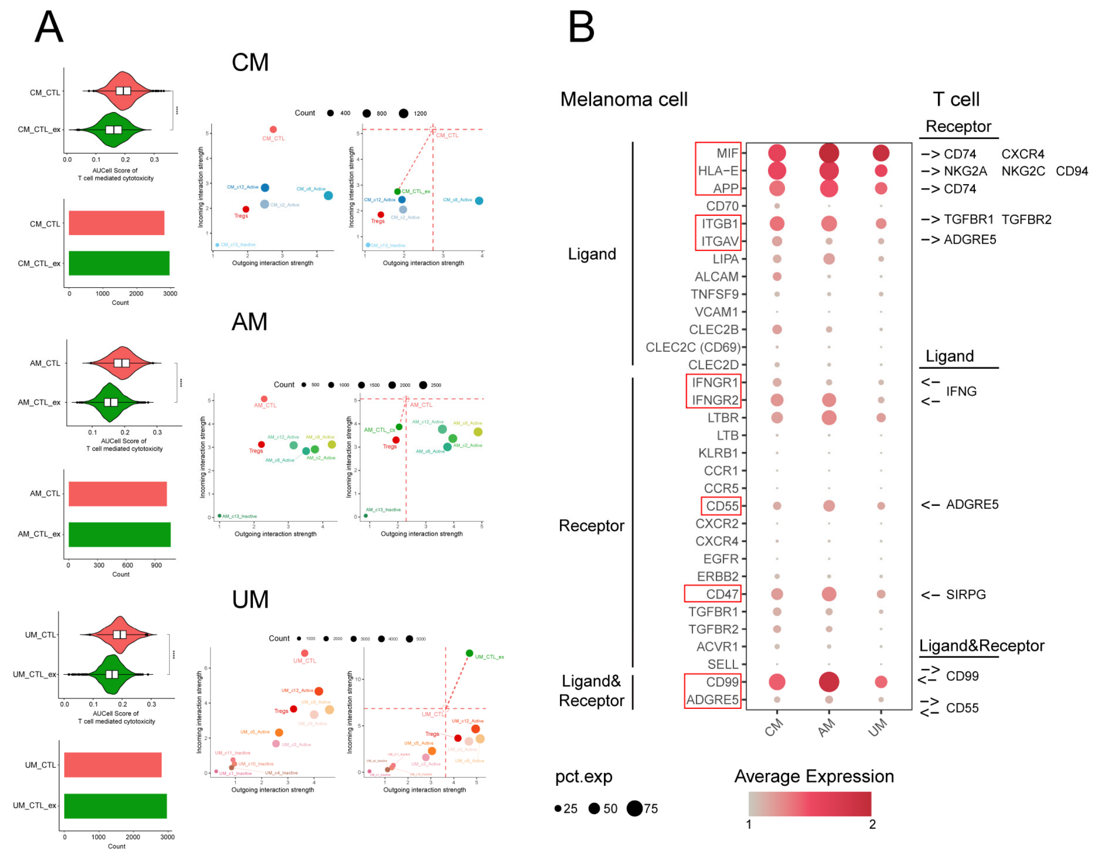

Figure S10 Communication patterns between tumor cells and T cells in three locations. A Violin plots showing the cytotoxicity score of CTL and CTL_ex in CM, AM and UM. Significance was determined using a two-sided, unpaired Wilcoxon rank-sum test, p-value denoted as *, ****p <= 0.0001. Bar plots showing the number of interactions of the inferred cell-cell communication from CTL, CTL_ex and melanoma tumor cell. Identify cell populations with significant changes in sending or receiving signals in each melanoma site (right panel). **B** Dot plot showing the expression of ligand and receptor genes (differ in communication between CTL, CTL_ex and tumor cells) in tumor cell from each melanoma site.

Figure S11

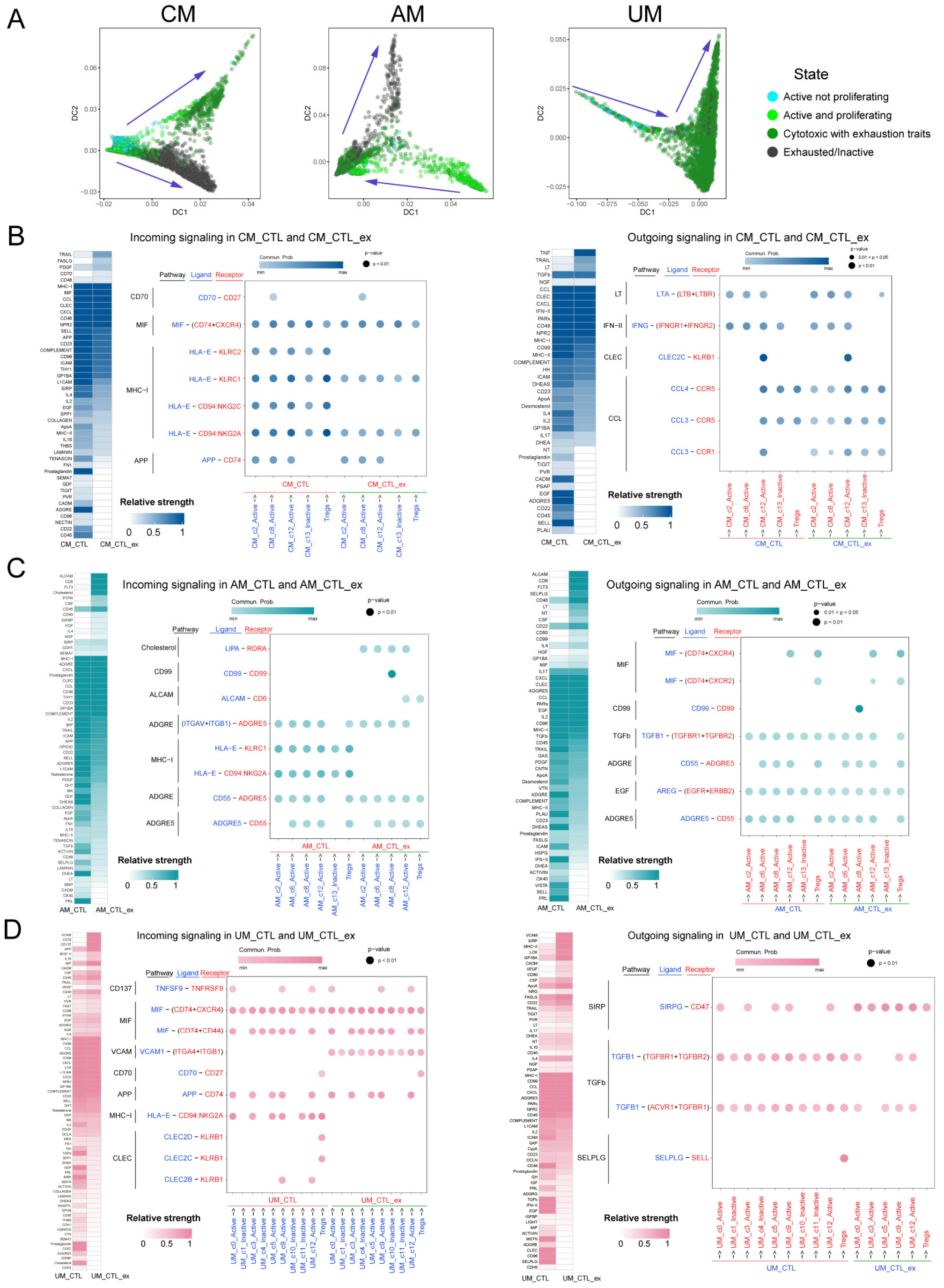

Figure S11 Incoming signaling and outgoing signaling of communication between CTL, CTL_ex and tumor cells. A Two-dimensional representation of T cell states along a pseudotemporal trajectory, inferred using Destiny. **B-D** Pathways of communication between CTL, CTL_ex and tumor cells in CM (**B**), AM (**C**) and UM (**D**) (left panel). Up-regulated and Down-regulated signaling between CTL, CTL_ex and tumor cells in CM (**B**), AM (**C**) and UM (**D**) (right panel).
